# Glycerol-Based Retrievable Heterogeneous Catalysts for Single-Pot Esterification of Palm Fatty Acid Distillate to Biodiesel

**DOI:** 10.3390/molecules27207142

**Published:** 2022-10-21

**Authors:** Balkis Hazmi, Mahnoush Beygisangchin, Umer Rashid, Wan Nur Aini Wan Mokhtar, Toshiki Tsubota, Ali Alsalme, Chawalit Ngamcharussrivichai

**Affiliations:** 1 Institute of Nanoscience and Nanotechnology, Universiti Putra Malaysia, Serdang 43400, Selangor, Malaysia; 2 Department of Chemical Sciences, Faculty of Science and Technology, Universiti Kebangsaan Malaysia, Bangi 43600, Selangor, Malaysia; 3 Department of Materials Science, Graduate School of Engineering, Kyushu Institute of Technology, 1-1 Sensuicho, Tobata-ku, Kitakyushu, Fukuoka 804-8550, Japan; 4 Chemistry Department, College of Science, King Saud University, Riyadh 1145, Saudi Arabia; 5 Center of Excellence in Catalysis for Bioenergy and Renewable Chemicals (CBRC), Faculty of Science, Chulalongkorn University, Bangkok 10330, Thailand; 6 Center of Excellence on Petrochemical and Materials Technology (PETROMAT), Chulalongkorn University, Bangkok 10330, Thailand

**Keywords:** biodiesel, sulfonated, glycerol, by-product, PFAD, heterogenous catalyst, esterification

## Abstract

The by-product of the previous transesterification, glycerol was utilised as an acid catalyst precursor for biodiesel production. The crude glycerol was treated through the sulfonation method with sulfuric acid and chlorosulfonic acid in a reflux batch reactor giving solid glycerol-SO_3_H and glycerol-ClSO_3_H, respectively. The synthesised acidic glycerol catalysts were characterised by various analytical techniques such as thermalgravimetric analyser (TGA), infrared spectroscopy, surface properties adsorption-desorption by nitrogen gas, ammonia-temperature programmed desorption (NH_3_-TPD), X-ray diffraction spectroscopy (XRD), elemental composition analysis by energy dispersive spectrometer (EDX) and surface micrographic morphologies by field emission electron microscope (FESEM). Both glycerol-SO_3_H and glycerol-ClSO_3_H samples exhibited mesoporous structures with a low surface area of 8.85 mm^2^/g and 4.71 mm^2^/g, respectively, supported by the microscopic image of blockage pores. However, the acidity strength for both catalysts was recorded at 3.43 mmol/g and 3.96 mmol/g, which is sufficient for catalysing PFAD biodiesel at the highest yield. The catalytic esterification was optimised at 96.7% and 98.2% with 3 wt.% of catalyst loading, 18:1 of methanol-PFAD molar ratio, 120 °C, and 4 h of reaction. Catalyst reusability was sustained up to 3 reaction cycles due to catalyst deactivation, and the insight investigation of spent catalysts was also performed.

## 1. Introduction

Rapid human population growth, industrialisation, and automation have led to an increase in energy demand, estimated to increase above 50% by 2030 [1]. Fossil fuels cannot compensate for the current consumption rate 105 times quicker than what nature creates [2]. In addition, fossil fuels are harmful to our environment through the emission of greenhouse gases and subsequent global warming [3]. Therefore, the study of clean energy has become the most overwhelming challenge. Following that, different alternative energy sources such as hydroelectric, solar, wind, geothermal, and biofuels are introduced and applied [2]. Among these energy sources, biofuels are seen as a fundamental tool for substituting fossil fuels due to their renewability [3].

Biofuels are kinds of fuels that include energy from geologically recent carbon fixation. They can be produced from starch, vegetable oils, animal fats, algal biomass, and waste biomass, which are renewable, non-toxic, and biodegradable [4]. Biodiesel is the most popular biofuel type produced from renewable sources with the same oil quality as diesel fuel [5]. Generally, biodiesel provides environmental benefits since its utilisation leads to decreased harmful emissions of CO_2_ with reduced greenhouse effects. Fundamentally, biodiesel can be achieved using catalysed triglycerides or free fatty acids (FFA) with short-chained alkyls such as methyl, propyl esters, and ethyl using esterification and trans-esterification [6]. Nevertheless, the significant disadvantage of using edible oil is related to the food safety issue, which makes the production cost less cost-effective. Therefore, nonedible feedstocks such as waste cooking oil, chicken fat, neem oil, palm fatty acid distillate (PFAD), etc, which are casually dumped into the environment, can be utilised for biodiesel production [7]. 

Commonly, biodiesel production needs to use an enzyme or catalyst (homogenous/heterogenous) to speed up the reaction. The process of biodiesel production is currently a big issue related to its production cost when applying homogenous acid catalysts such as sulphuric acid (H_2_SO_4_), hydrochloric acid (HCl), phosphoric acid (H_3_PO_4_), and nitric acid (HNO_3_) because of non-reusability and complex washing process for catalyst separation upon effecting the esterification reaction [8]. Currently, a heterogeneous catalyst is much more performable than a homogenous catalyst owing to its easy separation and low cost [9]. Heterogeneous catalysts are produced with primary acid or acid-basic groups regarding the nature of feedstocks, such as Karanja oil, waste cooking oil (WCO), palm fatty acid distillate (PFAD), animal fat (AF) and sludge oil [10]. These materials include a large amount of FFA, which is improper for primary catalyst use because it causes saponification problems [11]. Therefore, the use of solid acid catalysts such as sulfonated carbon [12] and sulphated metal oxide [13] are more feasible.

Researchers worked on the carbon-based catalyst as promisingly solid acid catalysts such as sulfonated carbohydrate-based catalysts [14,15,16,17,18], sucrose-derived carbon catalysts [12,19], *Jatropha curcas* biomass carbon catalysts [11,20] owing to their remarkable properties such as low cost, abundantly available, high porosity, and high surface area. The catalyst was reused 7 times, and catalyst activity was reported as >96% of FAME for the first three runs. Lokman et al. [21] prepared the esterification of PFAD by a sulfonated-glucose solid acid catalyst. They achieved 92.4% FAME yield at an optimum condition such as reaction temperature (65 °C) and reaction time (2 h and 15 min) reaction time. Furthermore, Thushari et al. [22] studied and reported esterification of waste palm oil 92.7% of FAME yield at optimum condition and 12 h using the catalytic activity of sulfonated activated carbon of coconut waste. 

In every transesterification reaction, 10 wt.% of the total end product would be glycerol as a by-product [23], and the total global production in 2020 showed a record of 4 billion litres [24]. The huge amount of glycerol from biodiesel can be used as feedstock for acrolein production via dehydration which is likely to be a part of problem-solving in managing an excessive amount of crude glycerol and reducing production costs from utilising pure glycerol [25]. Furthermore, glycerol can be employed as catalyst support since it contains a carbon chain [25] that can be functionalised with sulfuric acid for instance. A study by Shatesh et al. reported that in-situ carbonisation and sulfonation of glycerol successfully esterified 97.8% of biodiesel from PFAD at optimum parameters such as methanol to PFAD molar ratio (18:1), catalyst (5 wt.%), reaction temperature (90 °C) within 1 h of reaction [10]. Therefore, crude glycerol disposal and its application have become a thoughtful dilemma and economic and environmental liability for the biodiesel industry.

In this study, transesterification by-product glycerol has been transformed into acidic glycerol catalysts for the esterification of palm fatty acid distillate (PFAD) which possessed good catalytic activity and catalyst recovery. The acidic catalyst samples were prepared via the sulfonation process of sulfuric acid and chlorosulfonic acid, which were then known as glycerol-SO_3_H and glycerol-ClSO_3_H catalysts, respectively. The details of physical and chemical analysis of the prepared sample were carried out by different analysis techniques such as thermal degradation, surface textural and morphologies, total acidity strength, X-ray diffraction analysis, and elemental composition. In addition, catalytic optimisation was performed to determine the ideal factor of esterification, such as catalyst loading (wt.%), reaction time, methanol to PFAD molar ratio, and reaction temperature, which was then applied for catalyst reusability investigation. In addition, the factor of catalyst deactivation was studied. 

## 2. Results and Discussion

### 2.1. Thermal Degradation Analysis

**Figure 1:**
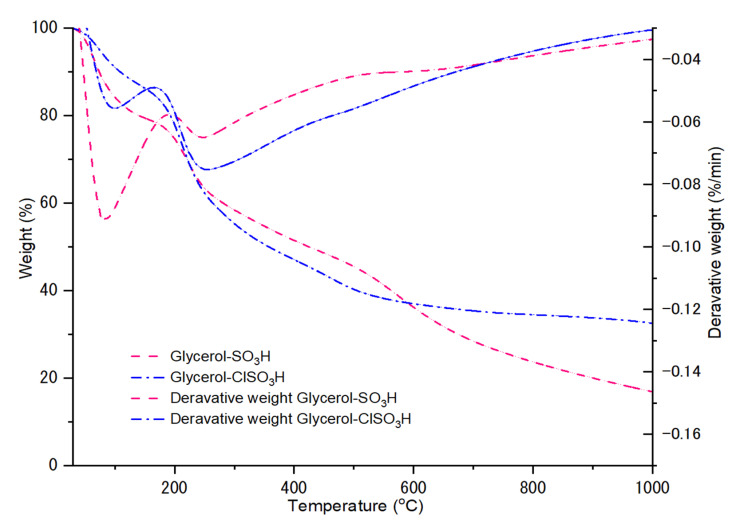
Thermal decomposition analysis of glycerol-SO_3_H and glycerol-ClSO_3_H catalyst.

The thermal decomposition and stability of acidic glycerol samples were evaluated by thermalgravimetric analyser from 30 °C to 1000 °C, as shown in Figure 1. The first thermal phase was determined as dehydration zone of adsorbed water and volatile organic matters up to 150 °C with weight loss of 13.1% (glycerol-ClSO_3_H) and 20.6% (glycerol-SO_3_H) from the catalyst’s surface. Second degradation zone was determined as the decay zone of sulfonate species found on the glycerol surface, which occurred at temperature range of up to 487 °C. In that phase, the weight lost for glycerol-SO_3_H and glycerol-ClSO_3_H were recorded at 31.6% and 39.8%, respectively. An extended decomposition temperature was recorded for the glycerol-ClSO_3_H catalyst sample at a maximum temperature of 589 °C, with 9.9% weight loss due to macromolecule bonding between chloride (Cl^−^) and sulphate (SO_3_^2−^) species [26]. Further weight loss from 589 °C to 1000 °C was denoted as the formation of solid residue.

### 2.2. Acid Properties Analysis

**Figure 2:**
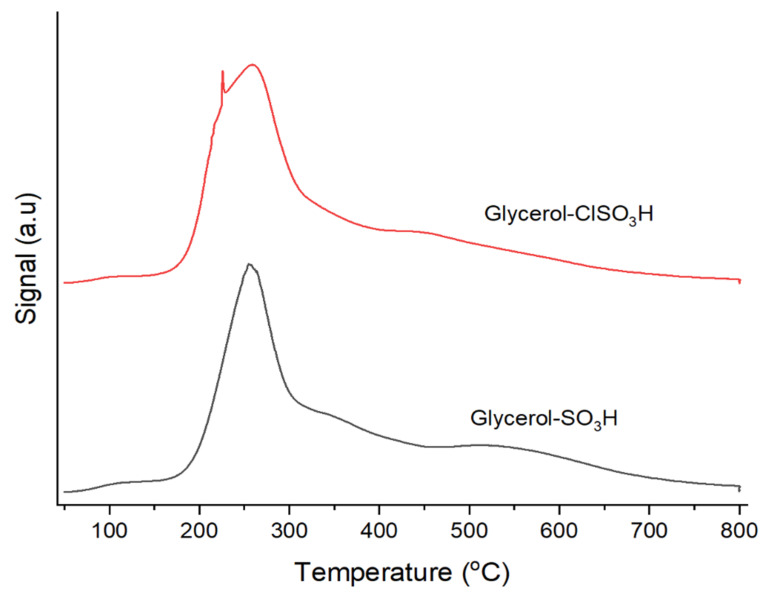
NH_3_-TPD absorption curves of acidic catalyst, glycerol-SO_3_H and glycerol-ClSO_3_H.

Figure 2 displayed the evaluation of solid glycerol catalysts for acid properties by NH_3_-TPD method and the amount of total acidity strength as tabulated in Table 1. A sharp and intense peak presented at a low-temperature range of 150–450 °C, possessing the weak Brönsted acid sites that consist of phenolic (-OH) and carboxylic groups interacted during adsorption-desorption with ammonia gas [27]. The interaction between NH_3_ and SO_3_H/ClSO_3_H species demonstrated a desorption curve such as strong Brönsted acid sites that occurred at temperatures 450–700 °C, respectively [28]. Therefore, the sulfonation of glycerol was successful with the presence of strong desorption at 450–700 °C by giving total acidity strength of 3.43 mmol/g and 3.96 mmol/g for glycerol-SO_3_H and glycerol-ClSO_3_H, respectively. A slight difference of acidity desorption would result in different efficiency of catalytic esterification. These findings were in agreement with FTIR and XRD results below which verified the impregnation of -SO_3_H and -ClSO_3_H group on the surface of glycerol. 

### 2.3. Functional Groups of Glycerol-SO_3_H and Glycero-ClSO_3_H

**Figure 3:**
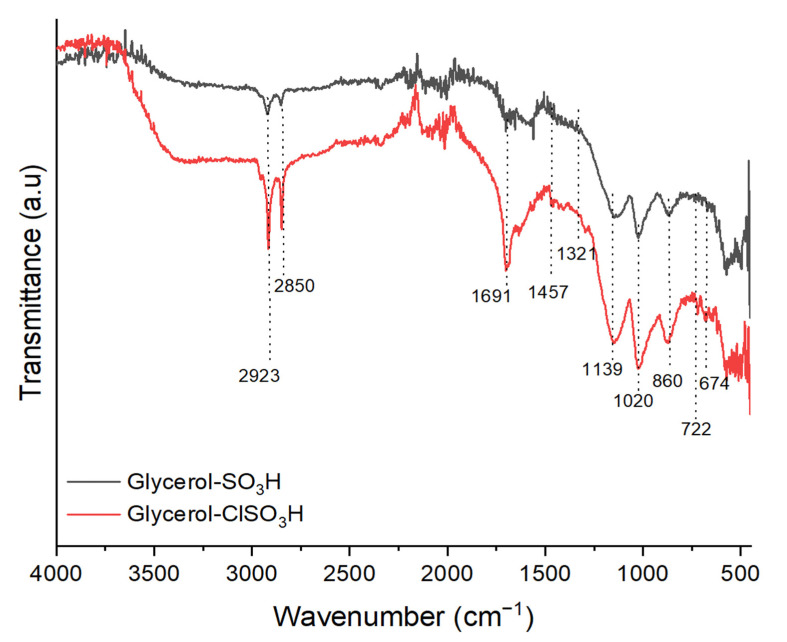
Infrared absorption spectra of glycerol-SO_3_H and glycerol-ClSO_3_H catalyst.

Figure 3 reveals the infrared absorption spectra of glycerol in samples that scanned from 4000 to 400 cm^−1^ at room temperature. Infrared vibration absorptions of C-H stretching were displayed at 2923 cm^−1^ and 2850 cm^−1^, indicating the asymmetric CH_2_ and symmetric CH_2_ stretching mode, respectively [29]. An intense IR absorption spectrum at 1691 cm^−1^ was assigned to C=O absorption band [30]. The absorption band for CH_2_ scissor bending appeared at 1457 cm^−1^ while 1321 cm^−1^ absorption spectrum was identified as C-H bending of aldehyde group [31]. The sulfonated group for both samples were determined at 1139 cm^−1^ and 1020 cm^−1^ of symmetrical and unsymmetrical stretching vibration of O=S=O [32], respectively. A single absorption band at 860 cm^−1^ showed the presence of OH bonding group of SO_3_H [33]. Furthermore, the stretching of S-O and C-S groups were exhibited at wavenumber of 722 cm^−1^ and 674 cm^−1^, respectively [34]. In addition, glycerol-ClSO_3_H sample showed highly intense spectra of O=S=O absorption due to the presence of Cl group that contributed to high electronegative than glycerol-SO_3_H.

### 2.4. Surface Area and Pore Size Analysis

**Figure 4:**
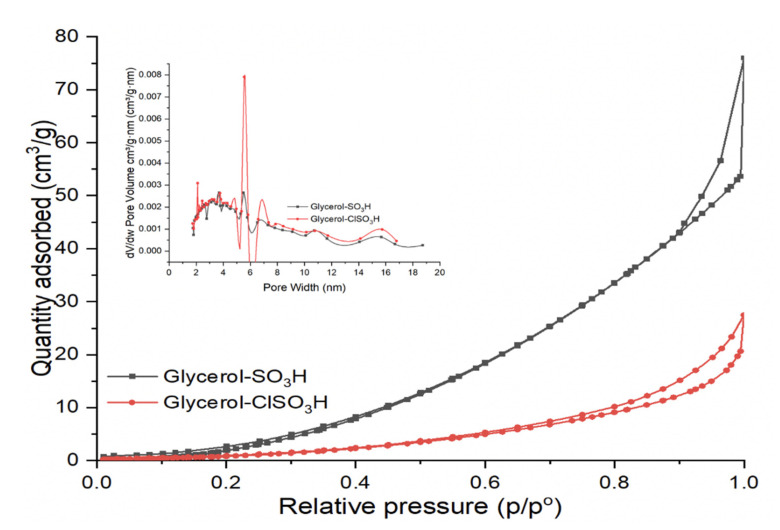
N_2_ adsorption-desorption surface and pore diameter distribution of produced Glycerol-SO_3_H and Glycerol-ClSO_3_H catalyst.

Surface textural analysis of synthesised glycerol-SO_3_H and glycerol-ClSO_3_H catalysts were measured as shown in Figure 4 and Table 1. According to Figure 4, Both samples seemed to possess type-IV isotherm patterns of mesoporous pore structures. Those mesoporous structures were classified as H_2_ hysteresis loops of disordered pores with pores blocking [35]. Low surface area of 8.85 m^2^/g (glycerol-SO_3_H) and 4.71 (glycerol-ClSO_3_H) depicted the solidification of glycerol through in-situ acid dehydration/sulfonation made of void and blockage pores which less adsorption-desorption of N_2_ on the catalyst surface. Pore size distribution analysis found that the catalyst samples consisted of bimodal pore distribution in mesoporous range, enhancing the diffusion of fatty acids on the catalyst’s active sites for esterification [36]. By the approaches from Barrett, Joyner and, Halenda (BJH), the mean pore diameter was calculated at 5.28 nm. 

### 2.5. X-ray Diffraction Analysis

**Figure 5:**
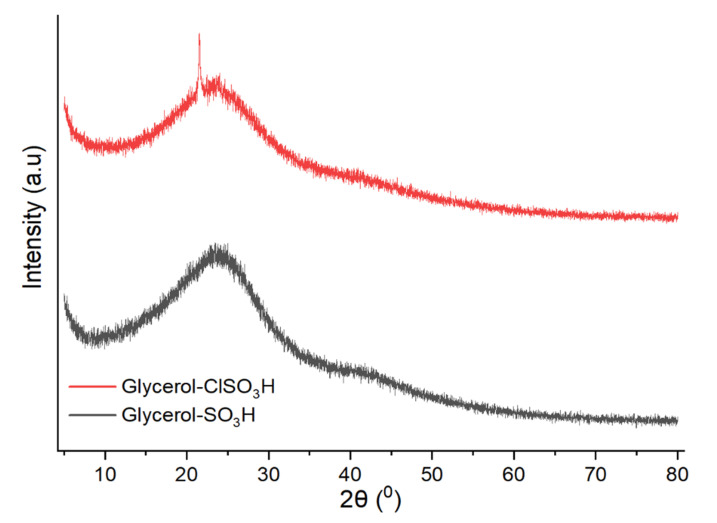
XRD signal analysis of acidic glycerol catalyst samples.

The XRD patterns for glycerol -SO_3_H and glycerol -ClSO_3_H catalyst are shown in Figure 5. Both samples exhibited two broad diffraction signals at 2θ angles of 10–32° and 40° to 50° that representing the unorganised order of polycyclic aromatic of amorphous carbon structure [33]. According to Sangar et al., the amorphous carbon structure could contribute to the esterification of fatty acids [11] by providing pores containing an active site for the reaction to occur. 

### 2.6. Microscopic Surface Morphology of Acidic Glycerol Catalysts

**Figure 6:**
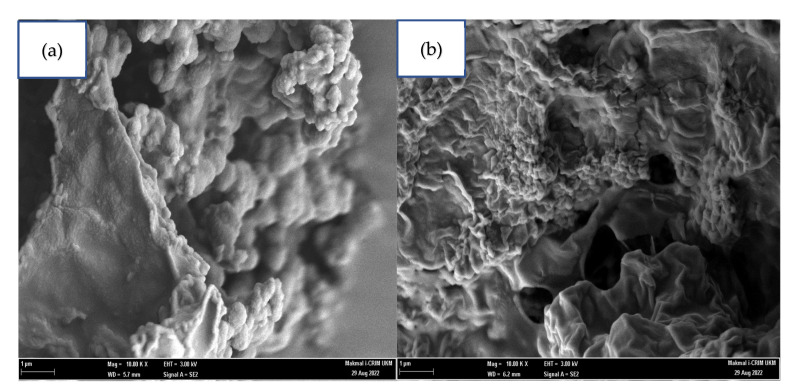
FESEM image under 10 K magnification power of (**a**) Glycerol-SO_3_H, (**b**) Glycerol-ClSO_3_H.

FESEM examined surface morphologies of the synthesised acidic glycerol catalysts at magnification power of 10 K (Figure 6). The micrograph images showed irregular pore patterns and unorganised surface covered with sulfonate and chloro-sulfonate species. As a result of sulfonation, the size of pore opening channels reduced and collapsed, in agreement with BET surface area and pore diameter findings. Lin et al. [37] and Pan et al. [38] also reported a similar structure modification of post-sulfonated catalyst samples. Both samples were contained of carbon, oxygen, and sulphur (chloride for glycerol-ClSO_3_H) as associated due to sulfonation. In addition, Table 1 presents the characterisation of the elemental composition by EDX. 

### 2.7. Catalyst Activity: Experimental Optimization Studies

#### 2.7.1. Catalyst Loading

**Figure 7:**
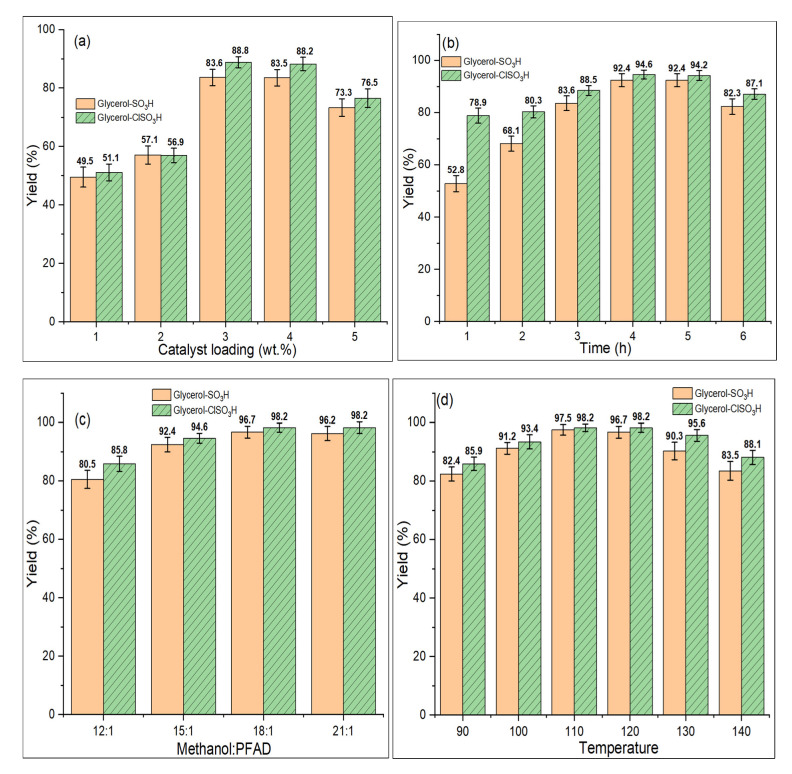
Catalytic performance optimization test (**a**) catalyst loading, (**b**) reaction time, (**c**) methanol-oil molar ratio and (**d**) reaction temperature.

Figure 7a presented the effect of catalyst loading concerning biodiesel yield at uniform reaction parameters (3 h, 15:1 methanol to PFAD molar ratio and 120 °C of esterification temperature). The reaction plots showed that as catalyst loading increases, the number of active sites engaging for the esterification increases, improving biodiesel yield [39]. The yield difference by both catalysts was significantly influenced by the active sites’ acidity strength, given that higher acidity content will esterify higher yield. The active sites were involved in converting the methanol into methoxide species [40] that were used for reacting with fatty acids of PFAD. The highest yield obtained was 83.6% and 88.8% when utilised 4 wt.% of glycerol-SO_3_H and glycerol-ClSO_3_H, respectively. However, yield reduction was found (73.3% and 76.5%) for the reaction of 5 wt.% catalyst loading due to mass transfer difficulty initiated for reversable reaction, as discussed by Ma et al. [27]. Therefore, 3 wt.% of catalyst loading was selected for further esterification reaction.

#### 2.7.2. Reaction Time

Figure 7b demonstrated the yield of esterified PFAD that significantly increased with the reaction time from 1 h to 4 h at constant 3 wt.% catalyst loading, 15:1 methanol to PFAD molar ratio and 120 °C of esterification temperature. The highest esterified yield obtained by glycerol-SO_3_H and glycerol-ClSO_3_H catalysts were 92.4% and 94.6%, respectively. During 4 h of reaction, the reactants (methanol and free fatty acids) gained sufficient time for adsorbing and desorbing due to optimised effective collision on catalyst active sites promoting homogeneity that enhancing towards the formation of methyl esters [41,42]. However, there were no considerable changes in biodiesel yield beyond 4 h of catalytic activity.

#### 2.7.3. Methanol-PFAD Molar Ratio

The effect of methanol to PFAD molar ratio on the efficiency of esterification was performed from 12:1 to 21:1 under consistency 3 wt.% of loaded catalyst, 3 h of reaction and 120 °C. The biodiesel yield increased with the methanol ratio, as revealed in Figure 7c, because at high methanol content, the esterification equilibrium constant was achieved for promoting methyl esters formation [43,44], which was recorded from ~80% to 98% (12:1–18:1). Meanwhile the biodiesel yield remained constant at 96.2% and 98.2% when esterified with 21:1 of methanol. Hence, 18:1 methanol to PFAD molar ratio was chosen for the esterification optimisation tests. 

#### 2.7.4. Reaction Temperature

The study of adjusted reaction temperature (90–120 °C) in response to biodiesel yield was carried out as shown in Figure 7d. At a reaction temperature of 90 °C, the obtained yields were 82.4% and 85.9%, then gradually inclined up to 97.5% and 98.2% at optimized temperature of 120 °C. Literally, PFAD esterification required high temperature to provide sufficient energy for converting its solid form into liquid, which will provide homogeneity with methanol for the reaction to occur. However, the yields dropped to 6–10% when the reaction temperature raised to 130 °C and 140 °C due to the increment of addition effective collision frequency (beyond optimized temperature) between PFAD-methanol mixture on the catalyst surface [33]. Thus, enhancing the effective collision between the methanol-PFAD phase with the acidic sites of glycerol catalysts for producing biodiesel at maximum yield of ideal temperature at 120 °C [45].

### 2.8. Plausible Reaction Mechanism of PFAD Esterification with Produced Acidic Glycerol in Presence of Methanol

**Figure 8:**
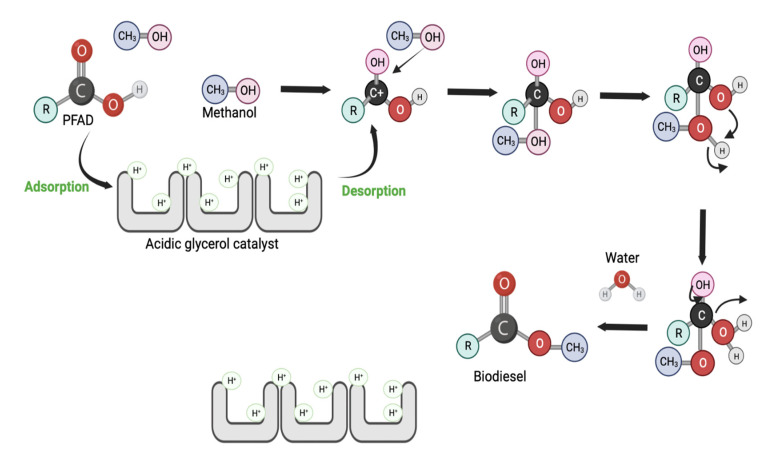
Proposed mechanism of PFAD-methanol esterification with acidic glycerol.

This study, the catalytic esterification reaction was drove by sulfonate acidic group impregnated on glycerol carbon-based surface as revealed in proposed mechanism in Figure 8. As the PFAD-methanol solution mixed with sulfonate glycerol catalyst, free fatty acid (FFAs) carbonyl oxygen atom interacted with H⁺ species of sulfonate group forming high electron affinity of carbonyl carbon atom. Next, promoting the nucleophilic attack from methanol (nucleophilic reaction) to interact with protonated fatty acids molecule, followed by proton transfer. Lastly, the PFAD biodiesel was obtained through series of FFAs restructure and proton elimination (water as by-product). 

### 2.9. Reusability and Characterization of Spent Catalysts

**Figure 9:**
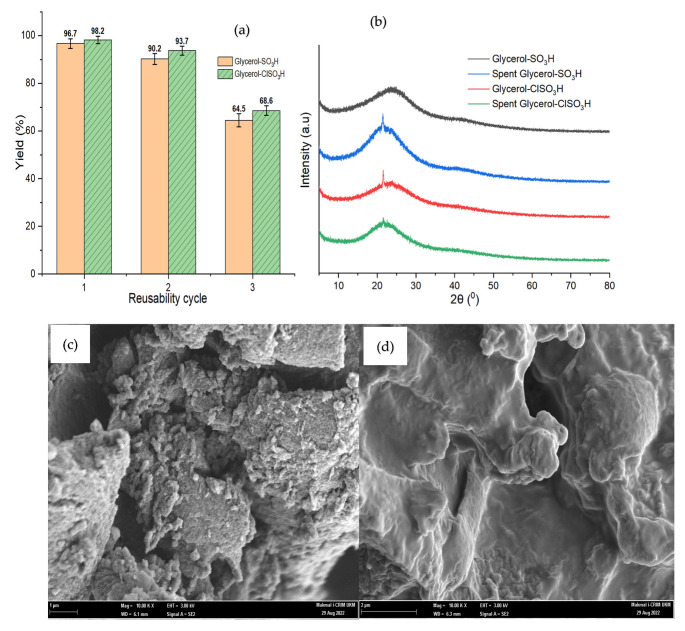
(**a**) Esterification reusability cycles graph, (**b**) XRD, (**c**) spent Glycerol-SO_3_H, (**d**) spent Glycerol-ClSO_3_H.

The acidic glycerol catalysts were retrieved by filtration and reused for the esterification of PFAD at optimal reaction conditions. Figure 9a shows the gradual reduction of biodiesel yield, which is significantly related to catalyst deactivation in the 3rd reaction cycle. The characteristics of the poor catalytic performance were studied by physical and chemical characterisation analysis. The diffraction patterns in Figure 9b revealed the intensity increment of spent catalysts at an amorphous region of 2θ = 15–32° due to carbon structural rearrangement of losing active components (-SO_3_H or ClSO_3_H) from the catalyst surface [21]. The loss of active sites mainly caused by adsorbed fatty acids that covered on catalyst surface directly reduced the surface area as depicted in Figure 9c and Table 1 thus hindered catalytic performance. Table 1 of further investigation of spent catalysts through acidity strength proved that total adsorption of NH_3_ was reduced as well as elemental composition of the active component such as S and Cl, hence lowering the biodiesel yield. 

### 2.10. Comparison Study of the Esterification by the Sulfonated Catalyst for PFAD Biodiesel Production

The comparison results of sulfonated catalysts for catalytic esterification of PFAD biodiesel are listed in Table 2. The yield of PFAD catalysed by sulfonated glycerol (Glycerol-SO_3_H and Glycerol-ClSO_3_H) were equal or higher over most catalysts. The catalyst efficiency was influenced by its physical and chemical properties in enhancing maximum biodiesel yield production. Room for improvement for the current synthesised catalyst is required for effectively esterified PFAD in shorter reaction time, low energy consumption, and high stability for catalyst reutilisation. 

## 3. Materials and Methods

### 3.1. Chemical Reagents

Sulfuric acid (H_2_SO_4_, 98%), chlorosulfonic acid (ClSO_3_H, 99%), methanol (CH_3_OH, 95%), *n*-hexane (C_6_H_14_), methyl heptadecanoate (99%) were analytical grade and used without purification. Palm fatty acid distillate (PFAD) was obtained from Sime Darby Sdn Bhd. Malaysia.

### 3.2. Preparation of Acidic Glycerol Catalysts

The obtained glycerol from previous transesterification reaction was separated from biodiesel and catalyst. 5 g of glycerol was weighed and transferred into a round bottom flask. 25 mL of concentrated sulfuric acid was added into the flask, and the reaction mixture was refluxed at 150 °C and stirred for 1 h under nitrogen gas flow. A bubbling effect was presented during the reaction of glycerol and sulfuric acid and was let to ease. The sulfonated glycerol was filtered and washed with distilled water to remove access sulfuric acid. The catalyst sample was overnight dried in the oven at 105 °C and kept in a screw cap sample bottle with a label of glycerol-SO_3_H. The same procedures were repeated by reacting the glycerol with concentrated chlorosulfonic acid to obtain a glycerol-ClSO_3_H catalyst. Both catalysts were applied for the esterification of PFAD.

### 3.3. Characterizations

Powder X-ray diffraction analysis was applied to access the phase structures of the synthesised acidic glycerol catalysts using Rigaku SmartLab XRD equipped with Hypix-400 Cu-Kα radiation, operated at room temperature with 40 kV and 30 mA radiation power. The textural analysis of degassed acidic glycerol samples was measured using Micrometrics ASAP 2020 at cryogenic nitrogen gas temperature (77 K). The catalyst’s degradation phases, and stability were evaluated using a thermogravimetric analyser, PerkinElmer Pyris Diamond. An approximate 4 mg of catalyst samples were degraded at a temperature range of 30 °C and 1000 °C with a temperature rate of 10 °C/min and 50 mL/min of N_2_ flow. An attenuated total reflection Fourier transform infrared spectrometer (ATR-FTIR, Nicolet iS10 FTIR) was utilised for determining the acidic catalyst sample’s functional groups over a scanning range of 400–4000 cm^−1^ with 64 scans and 4 cm^−1^ resolution. The surface morphology of synthesized acidic-glycerol samples was characterized via field emission scanning electron microscope, Zeiss-Merlin Compact, under a magnification of 10 K. The solid sample was previously coated with iridium. The elemental analysis was scanned by Oxford instrument equipped with the Zeiss-Merlin Compact. The acidity chemisorption of the catalysts was determined by ammonia-temperature programmed desorption (NH_3_-TPD, BELCAT II) equipped with a thermal conductive detector (TCD). The chemisorption was performed by purging NH_3_ absorption gas carried by He gas (30 mL/min) with a continuous heating process for the absorption from 50 °C to 800 °C. 

### 3.4. Esterification of Palm Fatty Acid Distillate (PFAD) Using Acidic Glycerol Catalysts

**Figure 10:**
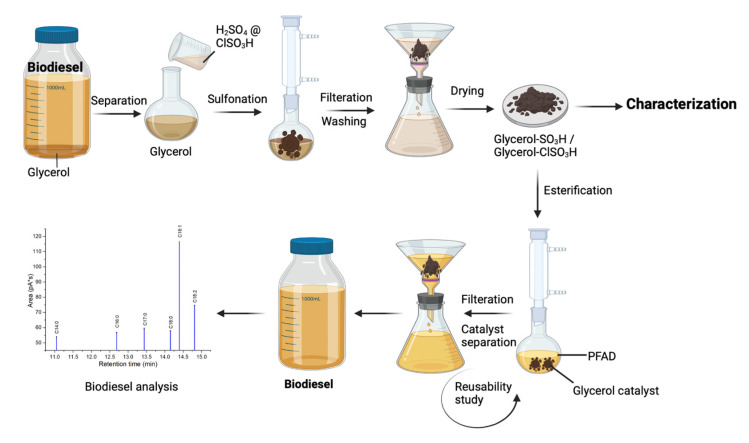
Process flow of catalyst preparation, esterification reaction and biodiesel analysis.

Optimisation reaction conditions for the esterification of PFAD and methanol catalysed by glycerol-SO_3_H and glycerol-ClSO_3_H catalyst were carried out by a reflux system equipped with cold condenser (±18 °C). In this work, reaction conditions were optimised as follows; catalyst loading (1–5 wt.%), reaction time (1–5 h), methanol to PFAD (MeOH:PFAD) molar ratio (12:1–21:1) and reaction temperature (90–120 °C) for each type of catalysts. At first, 10 g of PFAD was mixed with methanol (15:1) and 2 wt.% of acidic glycerol catalyst (glycerol-SO_3_H/glycerol-ClSO_3_H, respectively). Next, the mixture was stirred continuously for 3 h at a temperature of 120 °C. Upon the completion of esterification, the biodiesel product was separated from the catalyst and water through centrifugation. Each optimisation reaction was run three times, and the average value was calculated. The yield of biodiesel was analysed by gas chromatography. The complete process of catalysts synthesis and biodiesel production has been reported in the flowchart diagram (Figure 10). 

### 3.5. Biodiesel Yield Analysis

**Figure 11:**
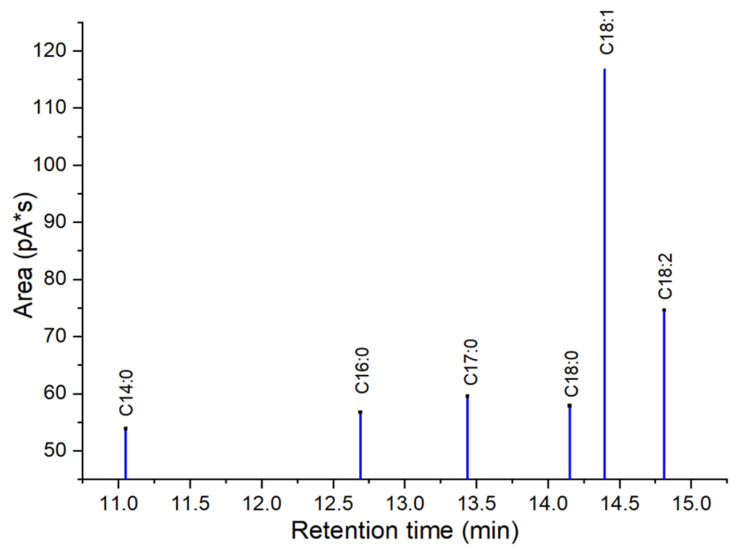
Chromatogram of methyl esters standard; C14:0 = methyl myristate, C16:0 = methyl palmitate, C17:0 = methyl heptadecanoate (internal standard), C18:0 = methyl stearate, C18:1 = methyl oleate, C18:2 = methyl linoleate.

The produced biodiesel was evaluated according to European standard method EN 14,103 by using gas chromatography (GC, Agilent 7890A) equipped with flamed ionization detector (FID). The viscous biodiesel sample was diluted with methyl heptadecanoate in n-hexane solution before subjecting for the GC injection (1 μL) which the injector was set at 250 °C. The separation of FAME components was detected through the elution on a polar capillary column (BPX-70, 60 mm × 0.25 mm × 0.25 mm) with oven temperature programmed of 100–250 °C at a ramp rate of 10 °C/min and hydrogen as carrier gas. The elution of methyl ester components was determined accordingly by retention time of the standard fatty acids as shown in Figure 11. The following formula calculated the FAME yield:

(1) $$Yield=\frac{Sum\;of\;esters\;peak-area\;of\;IS}{Area\;of\;IS}\times \frac{Concentration\;of\;IS\times Volume\;of\;IS}{Total\;weight\;of\;biodiesel}\times 100$$

*IS* = internal standard.

### 3.6. Catalysts Reusability

Catalytic reusability was studied by reusing the spent solid catalyst after each reaction cycle without catalyst reactivation which was carried out at their optimised conditions (3 wt.% of catalyst loading, 18:1 of methanol to oil molar ratio for 4 h of reaction at a temperature of 120 °C). The spent glycerol catalyst was collected through filtration and was washed with *n*-hexane to remove adsorbed oil, methanol, and other free fatty acids. Pre-washed spent catalysts were dried overnight before the esterification reaction. The catalytic tests were stopped when the achieved yield was reduced by 15–20% from the previous reaction’s yield. The spent catalysts were then characterized for deactivation study.

### 3.7. Statistical Analysis

All optimization studies of PFAD esterification have been analysed individually and the results were reported as mean ± standard deviation.

## 4. Conclusions

Current research discussed reutilizing glycerol from the transesterification by-product as an acidic catalyst for PFAD esterification. The treatment of glycerol with a strong acid such as sulfuric acid and chlorosulfonic acid created solid glycerol-SO_3_H and glycerol-ClSO_3_H catalysts. The properties and composition of solid sulfonated glycerol samples were characterised using different spectroscopic and analytical techniques. Thermal degradation analysis reported that the active groups of acid glycerol (-SO_3_H and ClSO_3_H) stability could retain up to 487 °C which can endure at high PFAD esterification reaction temperature. The availability of functional and active component groups and acidity strength were confirmed by infrared analysis, XRD and ammonia absorption. The infrared absorption bands related to sulfonate groups appeared at 1139 cm^−1^,1020 cm^−1^, 860 cm^−1^, 722 cm^−1^ and 674 cm^−1^ indicating the success in sulfonation treatment. Furthermore, the total acidity analysis recorded 3.43 mmol/g and 3.96 mmol/g of glycerol-SO_3_H and glycerol-ClSO_3_H catalyst, respectively, indicating the active acidic sites available for the esterification. The textural surface analysis by N_2_ physisorption and microscopic morphologies demonstrated uneven pore structures arrangement with pore blockage for glycerol-SO_3_H and glycerol-ClSO_3_H catalyst sample, resulting low surface area of 8.85 m^2^/g and 4.71 m^2^/g respectively. The synthesised acidic glycerol catalyst exhibited good catalytic performance with more 95% of biodiesel yield under ideal reaction conditions; 3 wt.% of catalyst loading, 18:1 methanol-oil molar ratio and 4 h reaction time at 120 °C of temperature. However, the effectiveness of the catalysts only sustained up to 3 reaction cycles due to the deactivation of catalyst attributed to pore blockage by unreacted fatty acid on the catalyst’s active sites, thereby reducing the number of active sites and declining the reaction rate for biodiesel production. Overall, by product such as glycerol (from transesterification) can be utilised as valuable material by converting into low-cost acidic catalyst, glycerol-SO_3_H and glycerol-ClSO_3_H for PFAD diesel.

## Figures and Tables

**Table 1:** Textural properties, elemental compositions, and total acidity of acidic glycerol catalysts.

Catalyst	BET Specific Surface Area (m^2^/g)	Pore Volume (cm^3^/g)	Average Pore Size (nm)	Total Acidity (mmol/g)	Element (wt.%)			
					C	O	S	Cl
Glycerol-SO_3_H	8.85 (1.01) *	0.07 (0.02) *	5.28 (5.02) *	3.43 (1.05) *	56.3 (66.1) *	25.7 (25.4) *	18.0 (8.5) *	- -
Glycerol-ClSO_3_H	4.71 (1.04) *	0.02 (0.01) *	5.28 (5.20) *	3.96 (1.37) *	48.9 (56.0) *	32.4 (32.1) *	16.6 (10.6) *	2.1 (1.2) *

* the values in the parentheses are for the spent catalysts.

**Table 2:** Comparison analysis of sulfonated catalyst properties and catalytic esterification performances.

Catalyst	BET Specific Surface Area (m^2^/g)	Acidity (mmol/g)	Reaction Parameters	ReusabilityCycle	Yield (%)	Ref.
HSO_3_/SnO_2_	7.50	5.30	4 wt.%, 100 °C, 3 h, 9:1	5	96.4	[46]
BSY-SO_3_H	889	0.58	8 wt.%, 65 °C, 3 h, 21:1	4	87.8	[39]
PSS-ICG	8.70	14.64	2.5 wt.%, 80 °C, 4 h, 10:1	-	96.3	[47]
EFB-4BDS	2.85	3.93	20 wt.%, 7 h	-	98.1	[48]
Glycerol-SO_3_H Glycerol-ClSO_3_H	8.85 4.71	3.43 3.96	3 wt.%, 120 °C, 4 h, 18:13 wt.%, 120 °C, 4 h, 18:1	3 3	96.7 98.2	This study This study

## Data Availability

Not applicable.
